# On the Use of Veratra in the Acute Articular Rheumatism of Children

**Published:** 1863-11

**Authors:** 


### ON THE USE OF VERATRA IN THE ACUTE ARTICULAR RHEUMATISM OF CHILDREN.

Acute articular rheumatism may be regarded as a disease of the sthenic order, and depressing remedies may therefore be employed in young subjects who are attacked by it, provided that these remedies are used discreetly, and that their effects are closely watched. M. Bouchut has made a number of practical observations on the employment of veratria in the treatment of acute articular rheumatism in children. He frequently, and indeed almost daily, employs this medicine in the disease alluded to, and he is well satisfied with the effects produced, both in regard to its tolerance by the patients and to its efficacy. He administers the veratria to children either in pills or in a julep, taking care to add an equal quantity of an opiate to facilitate tolerance. He begins by a dose of 1 to 5 milligrammes, (a milligramme is ·0154 of a troy grain,) according to the age, the last dose being that which he generally prescribes for a child at the age of ten. He then increases the dose progressively from day to day, doubling it the second day, tripling it the third, and so on in succession, but always watching the effects produced, and regulating the dose accordingly. He has thus sometimes reached thirty-five or forty milligrammes a day, given in seven or eight pills at equal intervals in the twenty-four hours. As soon as there is any sensible improvement in the pain and in the rate of the circulation, he follows an inverse progression, diminishing the dose every day, so as to return gradually to the original quantity, and at last discontinuing the use of the medicine altogether.—*Bulletin Gen. de Therapeutique*, *July* 15, 1862.

